# Macro-Nutrient Stoichiometry of Glacier Algae From the Southwestern Margin of the Greenland Ice Sheet

**DOI:** 10.3389/fpls.2021.673614

**Published:** 2021-06-28

**Authors:** Christopher J. Williamson, Thomas Turpin-Jelfs, Miranda J. Nicholes, Marian L. Yallop, Alexandre M. Anesio, Martyn Tranter

**Affiliations:** ^1^Bristol Glaciology Centre, School of Geographical Sciences, University of Bristol, Bristol, United Kingdom; ^2^School of Biological Sciences, University of Bristol, Bristol, United Kingdom; ^3^Department of Environmental Science, Aarhus University, Aarhus, Denmark

**Keywords:** glacier algae, C:N:P, Greenland Ice Sheet, stoichiometry, supraglacial

## Abstract

Glacier algae residing within the surface ice of glaciers and ice sheets play globally significant roles in biogeochemical cycling, albedo feedbacks, and melt of the world’s cryosphere. Here, we present an assessment of the macro-nutrient stoichiometry of glacier algal assemblages from the southwestern Greenland Ice Sheet (GrIS) margin, where widespread glacier algal blooms proliferate during summer melt seasons. Samples taken during the mid-2019 ablation season revealed overall lower cellular carbon (C), nitrogen (N), and phosphorus (P) content than predicted by standard microalgal cellular content:biovolume relationships, and elevated C:N and C:P ratios in all cases, with an overall estimated C:N:P of 1,997:73:1. We interpret lower cellular macro-nutrient content and elevated C:N and C:P ratios to reflect adaptation of glacier algal assemblages to their characteristic oligotrophic surface ice environment. Such lower macro-nutrient requirements would aid the proliferation of blooms across the nutrient poor cryosphere in a warming world. Up-scaling of our observations indicated the potential for glacier algal assemblages to accumulate ∼ 29 kg C km^2^ and ∼ 1.2 kg N km^2^ within our marginal surface ice location by the mid-ablation period (early August), confirming previous modeling estimates. While the long-term fate of glacier algal autochthonous production within surface ice remains unconstrained, data presented here provide insight into the possible quality of dissolved organic matter that may be released by assemblages into the surface ice environment.

## Introduction

Microbial communities that reside on the surfaces of glaciers and ice sheets play globally significant roles in carbon (C) and nutrient cycling and surface ice melt (Hodson et al., 2008; Stibal et al., 2012; Anesio et al., 2017; Williamson et al., 2018, 2020). Of particular importance are Streptophyte “glacier algae” (Williamson et al., 2019), whose presence in surface ice lowers the bare ice albedo, enhances solar energy absorption, and drives surface melt through the process of “biologically driven albedo reduction” (Yallop et al., 2012; Stibal et al., 2017; Williamson et al., 2018, 2020; Cook et al., 2020). On the surface of the Greenland Ice Sheet (GrIS), summer blooms of glacier algae are responsible for widespread albedo decline that has paralleled accelerating surface melt since the early 1990s (Yallop et al., 2012; Tedesco et al., 2016; Stibal et al., 2017; van den Broeke et al., 2017; Cook et al., 2020; Tedstone et al., 2020; Williamson et al., 2020), with recent estimates attributing an additional 5.5–8.0 Gt of runoff to glacier algal growth along the western ice sheet margin; 6–9% of the total runoff (Cook et al., 2020). Given that melt of the GrIS is the single largest cryospheric contributor to global eustatic sea level rise (Bamber et al., 2018), constraining bloom dynamics into the future remains a significant research priority (Williamson et al., 2019, 2020; Cook et al., 2020; Tedstone et al., 2020).

Glacier algal blooms initiate following snow line retreat (Williamson et al., 2018), with population doubling times on the GrIS ranging 3.75–5.5 days, and maximal cell densities approaching 10^5^ cells mL^–1^ of melt water during major bloom years (Yallop et al., 2012; Stibal et al., 2017; Williamson et al., 2018, 2020). At these cell densities, widespread albedo decline is driven by significant secondary phenolic pigmentation produced by glacier algae to protect their low-light adapted chloroplasts (Williamson et al., 2020). By dissipating the intercepted incident irradiance as heat, this secondary pigmentation also generates liquid water proximal to the cells, driving surface ice ablation while providing access to nutrient and other resources required to promote algal growth (Dial et al., 2018; Williamson et al., 2020). Accordingly, growth proceeds as a function of bare-ice melt duration, such that strong patterning in accumulated biomass is apparent across the ablation zone, with maximal cell densities accumulated within the most marginal regions that experience the longest ablation periods, and a decreasing trend toward the equilibrium line (Williamson et al., 2020). As the ablation period progresses into polar winter, the fate of accumulated biomass remains unknown, although glacier algal species have been shown to overwinter in alpine locations (Remias et al., 2012b), and active glacier algal assemblages have been observed in GrIS shallow surface ice prior to snow line retreat (Nicholes et al., 2019).

One of the biggest questions remaining about glacier algal blooms asks, What factors limit the distribution and magnitude of blooms in space and time? While physical conditions such as snow-pack height, light availability, and temperature produce a first order control on the ability of blooms to form and proliferate in any given year (Tedstone et al., 2017; Williamson et al., 2020), little information currently exists on potential “bottom-up” or “top-down” regulation of blooms in supraglacial environments, restricting abilities to project bloom occurrence into the future (Williamson et al., 2019; McCutcheon et al., 2021). Within the marine environment, for example, top-down pressures such as zooplankton grazing and/or bottom-up availability of nutrients represent fundamental controls on phytoplankton biomass (Huppert et al., 2002). Supraglacial environments are characterized by truncated, microbially dominated trophic structures (Anesio and Laybourn-Parry, 2012; Anesio et al., 2017) and highly oligotrophic conditions (Hawkings et al., 2016; Wadham et al., 2016), indicating that large potential exists for bottom-up limitation of glacier algal blooms.

To date, field observations of ambient hydrochemistry during bloom progression have demonstrated a bulk phase shift toward organic over inorganic nutrient resources within surface ice (Holland et al., 2019), which coupled with 28-times lower secondary production relative to primary production (Yallop et al., 2012; Nicholes et al., 2019) has been interpreted to imply inefficient remineralization of inorganic nutrient resources within GrIS surface ice, and a potential mechanism of bottom-up control (Nicholes et al., 2019). Recently, McCutcheon et al. (2021) provided the first evidence for such bottom-up control of glacier algal blooms on the GrIS, highlighting inorganic phosphorus (P_*i*_) limitation and the importance of locally sourced hydroxylapatite in supporting bloom proliferation, and likely important roles for heterotrophic bacterial and fungal communities in accelerating apatite weathering and thus P availability to glacier algal communities. This study did not, however, directly quantify glacier algal abundance within samples, precluding direct calculation of cellular elemental quotas. The importance of this information is illustrated by the findings of Holland et al. (2019) for the same bloom and sampling sites, who concluded that there was sufficient inorganic macro-nutrient availability within surface ice to support the magnitude of glacier algal bloom apparent during the 2016 ablation season when assuming Redfield stoichiometry for glacier algal C, nitrogen (N), and P requirements.

Fundamental to deciphering the importance of bottom-up controls on glacier algal blooms is therefore knowledge of the macronutrient cellular requirements of glacier algal cells. While Redfield stoichiometry provides an important framework against which ambient nutrient concentrations can be contrasted (Redfield, 1958), deviations from this ratio are well documented across several algal lineages (Hecky et al., 1993; Geider and La Roche, 2002; Quigg et al., 2003), with individual cell stoichiometry shown to be dynamic relative to a plethora of drivers (Geider and La Roche, 2002; Quigg et al., 2003; Dickman et al., 2006; Finkel et al., 2016). Here, we provide an estimate of glacier algal macro-nutrient (C, N, and P) cellular stoichiometry in assemblages sampled from the surface of the GrIS. A snapshot of glacier algae stoichiometry was determined for assemblages residing within surface ice sampled from the south western ice sheet margin during the 2019 ablation season in order to provide a first order approximation of the elemental requirements of glacier algal assemblages and to investigate the potential for divergence from Redfield dynamics. This data is important for efforts to project the occurrence of blooms into the future and to constrain bloom impacts to cycles of C and macro-nutrients.

## Materials and Methods

### Study Area and Sampling Details

Surface ice containing glacier algal assemblages was sampled across August 8–10 (total number of samples = 28) from the marginal ablation zone in the south western GrIS proximal to Point 660. For all samples, the top 2 cm of surface ice was sampled using a pre-cleaned ice saw, transferred into sterile Whirl-Pak^®^ bags (Madison, WI, United States), and melted slowly in the dark over 24 h at 4°C. The melted surface ice was subsequently homogenized and sub-sampled for further analyses including 1 mL sub-sampled into 15 mL centrifuge tubes and fixed immediately with 2% glutaraldehyde final concentration for subsequent algal cell enumeration; triplicate 100–200 mL subsamples filtered onto pre-combusted 25 mm diameter glass microfiber filters (0.7 μm retention; Thermo Fisher Scientific, Pittsburgh, PA, United States), frozen immediately at −20°C for subsequent determination of glacier algal C, N content; and duplicate 100–200 mL subsamples filtered onto pre-combusted 47 mm diameter glass microfiber filters (0.7 μm retention; Thermo Fisher Scientific, Pittsburgh, PA, United States), frozen immediately at −20°C for subsequent determination of glacier algal P content. All samples were transported back to the University of Bristol for subsequent processing.

### Algal Cell Enumeration and Cell Biovolume Estimation

Algal abundance (cells mL^–1^) was determined using methods described by Williamson et al. (2018) on 1 mL aliquots of melted surface ice using a modified Fuchs-Rosenthal hemocytometer (0.2 mm by 1/16 mm^2^; Hawksley, Lancing, United Kingdom) on a Leica M205 C stereomicroscope (Wetzlar, DE) with attached GXCAM HiChrome-Met HD microscope camera (GT Vision Ltd., Stansfield, United Kingdom). Glacier algal cellular biovolume (μm^3^) was determined from measurements of cell length and diameter taken using ImageJ software (version 1.52n), for which glacier algal cells were considered to be regular cylinders (Hillebrand et al., 1999).

### Algal Carbon and Nitrogen

Using methods outlined by Lorrain et al. (2003), the C (after the removal of carbonates by acid fumigation) and N content of glacier algae retained on 25 mm diameter glass microfiber filters (0.7 μm retention; Thermo Fisher Scientific, Pittsburgh, PA, United States), which had been freeze-dried over 24 h, were determined using an elemental analyzer (Elementar vario PYRO cube^®^, Langenselbold, Hesse, DE). The detection limits of elemental concentrations were 0.001% for both elements measured, and the coefficient of variation (CV) for C and N according to six replicates of an organic analytical standard (NC Soil Standard 338 40025, cert. 133317, C = 2.29%, N = 0.21%; Elemental Microanalysis Ltd., United Kingdom) were 5.60% and 2.74%, respectively. To determine the quantity of C and N associated with algal biomass (μg mL^–1^ of surface ice), the bulk glacier algal abundance (cells mL^–1^) was multiplied by the quantity of cellular C and N (μg cell^–1^).

### Algal Phosphorus

Concentrations of total P (P_*t*_) and P_*i*_ associated with glacier algae were determined using methods adapted from Hedley and Stewart (1982) and Stibal et al. (2008). Paired 47 mm diameter glass microfiber filters (0.7 μm retention) were added to separate pre-cleaned 15 mL polypropylene centrifuge tubes (Thermo Fisher Scientific), frozen at −80°C and subsequently freeze-dried for 24 h. After freeze-drying, 1 mL of ethanol-free chloroform (CHCl_3_; Thermo Fisher Scientific) was added to one from each pair of centrifuge tubes. These tubes were then agitated three times for 10 s using a vortex mixer at 15 min intervals before being placed under a fume hood for 24 h to allow the CHCl_3_ to evaporate. Both the unfumigated and CHCl_3_-fumigated filters from each pair were then amended with 6 mL (∼ 1:60 w/v retained sample:extractant) of 1 M magnesium chloride (MgCl_2_; Thermo Fisher Scientific), agitated at 200 rpm on a reciprocal shaker for 16 h and centrifuged at 1,217 × *g* at 0°C for 12 min. An aliquot of 1.5 mL from the resulting extracts was transferred to a 9 mL muffled digest tube, mixed with 0.5 mL of an oxidizing agent comprised of 9 g of NaOH and 40 g of potassium persulfate (K_2_S_2_O_8_; Thermo Fisher Scientific) in 1 L of Milli-Q^®^ water, and autoclaved for 60 min at 121°C (digested). The undigested and digested MgCl_2_ extracts pertaining to both the unfumigated and CHCl_3_-fumigated filters were then filtered to 0.45 μm using mixed cellulose ester membranes (Whatman^®^, Maidstone, United Kingdom). Concentrations of P_*i*_ and P_*t*_ were determined in the undigested and digested MgCl_2_ extracts, respectively, using a standard orthophosphate colorimetric technique on a photometric meter (Gallery^TM^ Plus Discrete Analyzer, Thermo Fisher Scientific, Waltham, MA, United States). The CV (six replicate mid-range standards) for P_*i*_ and P_*t*_ were ≤0.20%. In addition, the limits of detection (LoD; three times the standard deviation of six replicate method blanks) for P_*i*_ and P_*t*_ were 4.73 μg L^–1^ and 1.93 μg L^–1^, respectively. Samples were blank corrected when blank concentrations exceeded the detection limits. All reagents used were of analytical grade. Algal P_*i*_ and P_*t*_ were calculated, respectively, as the differences between P concentrations in the undigested and digested MgCl_2_ extracts for the CHCl_3_-fumigated and unfumigated filters within each pair.

### Ambient Chemistry

Concentrations of dissolved organic C (DOC) and dissolved total N (DTN) in filtered ice samples were determined on a total organic C analyzer (Shimadzu TOC-L_*CPH*_, Kyoto, Japan) coupled with a total N measuring unit (Shimadzu TNM-L). The ammonium-N (NH_4_^+^-N), nitrate-N (NO_3_^–^-N), and phosphate-P (PO_4_^3–^-P) contents of the same samples were quantified on a photometric meter (Gallery^TM^ Plus Discrete Analyzer). According to six replicate standards, the CV for the DOC, DTN, NH_4_^+^-N, NO_3_^–^-N, and PO_4_^3–^-P analyses were 3.71%, 7.38%, 0.20%, 0.26%, and 0.35%, respectively. The corresponding detection limits for the same analyses were 51.40 μg L^–1^, 39.12 μg L^–1^, 1.19 μg L^–1^, 3.13 μg L^–1^, and 4.95 μg L^–1^. Dissolved organic N (DON) was calculated for each sample as the difference between concentrations of DTN and dissolved inorganic N (DIN; sum of NH_4_^+^-N and NO_3_^–^-N).

### Data Analyses

All plots and statistical analyses were performed using R version 3.4.1 in accordance with methods outlined by Crawley (2005).

## Results

### Ambient Chemistry of Surface Ice

Major C and macro-nutrient phases were determined for GrIS surface ice from which glacier algal assemblages were sampled to provide contextual hydrochemistry information to contrast with glacier algal cellular stoichiometries. Dissolved nutrient phases measured from melted surface ice were dominated by organic phases (Table 1), with the majority comprised of DOC, which exhibited concentrations ∼ 18 times greater than DON. In contrast, the concentrations of dissolved inorganic nutrients fell at or below the LoD. Dissolved inorganic N was only measurable in the form of NH_4_^+^ and accounted for ∼ 4% of the DTN, while PO_4_^3–^-P concentrations were below the LoD in all cases.

**TABLE 1:** Concentrations of dissolved organic carbon (DOC), total dissolved nitrogen (DTN), organic nitrogen (DON), ammonium-nitrogen (NH_4_^+^-N), nitrate-nitrogen (NO_3_^–^-N), and phosphate-phosphorus (PO_4_^3–^-P) in surface ice from the south western margin of the Greenland Ice Sheet (GrIS) during the 2019 ablation season (mean ± standard error, *n* = 12).

DOC (μg L^–1^)	DTN (μg L^–1^)	DON (μg L^–^^1^)	NH_4_^+^-N (μg L^–1^)	NO_3_^–^-N (μg L^–1^)	PO_4_^3–^-P (μg L^–1^)
1,964 ± 162	113 ± 27	108 ± 27	2.8 ± 1.4	1.6 ± 1.1*	0.9 ± 0.4*

*Below limit of detection (three times the standard deviation of six replicate method blanks).

### Assemblage Characteristics

**FIGURE 1:**
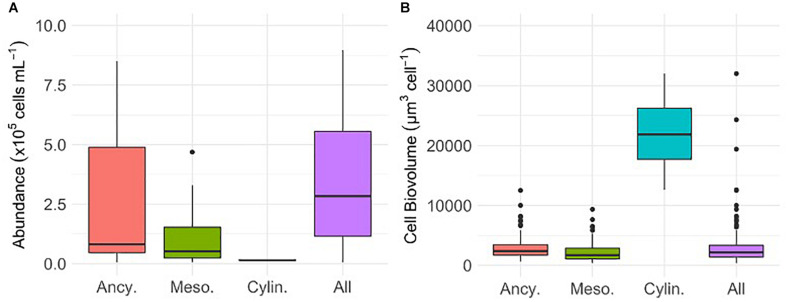
The abundance **(A)** and cellular biovolume **(B)** of *Ancylonema* cf. *nordenskiöldii* (Ancy., *n* = 19 abundance, *n* = 215 biovolume), cf. *Mesotaenium berggrenii* (Meso., *n* = 20 abundance and *n* = 140 biovolume), *Cylindrocystis brebissonii* (Cylin., *n* = 2 abundance, *n* = 5 biovolume), and the total glacier algal assemblage (All, *n* = 21 abundance *n* = 360 biovolume) sampled in surface ice from the south western margin of the Greenland Ice Sheet (GrIS) during the mid-2019 ablation season. Plots show median ± interquartile range.

Glacier algal assemblages ranged in abundance from 0 to 8.97 × 10^5^ cells mL^–1^ and were comprised predominantly of *Ancylonema* cf. *nordenskiöldii* (67.9%) and cf. *Mesotaenium berggrenii* (31.7%; Figure 1A). Mean cellular biovolumes for *A.* cf. *nordenskiöldii* and cf. *M. berggrenii* were 2,788 ± 121 μm^3^ cell^–1^ and 2,197 ± 131 μm^3^ cell^–1^, respectively (Figure 1B). In contrast, fewer than 1% of cells were identified as *Cylindrocystis brebissonii*, though mean cellular biovolumes of these cells were an order of magnitude larger than *A.* cf. *nordenskiöldii* and cf. *M. berggrenii* (Figure 1B).

**FIGURE 2:**
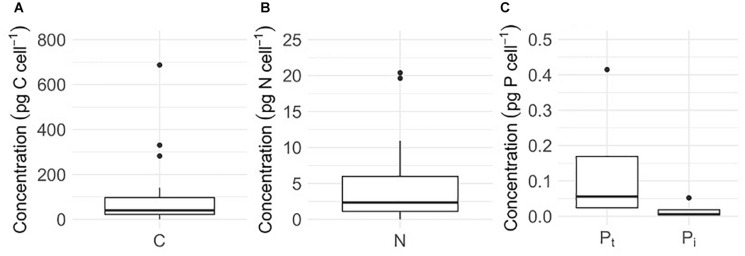
Cellular concentrations of **(A)** carbon (C, *n* = 28), **(B)** nitrogen (N, *n* = 28), **(C)** total phosphorus (P_*t*_, *n* = 4), and **(C)** inorganic phosphorus (P_*i*_, *n* = 4) of glacier algal assemblages comprised of *Ancylonema* cf. *nordenskiöldii*, cf. *Mesotaenium berggrenii*, and *Cylindrocystis brebissonii* in surface ice from the south western margin of the Greenland Ice Sheet (GrIS) during the mid-2019 ablation season. Plots show median ± interquartile range.

Cellular C and N contents of glacier algal assemblages averaged 106 ± 35 pg C cell**^–^**^1^ and 4.5 ± 1.3 pg N cell**^–^**^1^, respectively (Figures 2A,B), with cellular atomic C:N ratios ranging from 15.6 to 40.8. Using measured values of cellular C and N, we estimate an average of 27.4 ± 6.0 μg C mL**^–^**^1^ and 1.2 ± 0.25 μg N mL**^–^**^1^ to be contained within glacier algal biomass within the surface ice environment during our sampling period. In contrast to cellular C and N, quantifying algal P presented challenges as concentrations fell below the limit of detection (LoD), with P_*t*_ and P_*i*_ only detectable in seven and four of 28 samples, respectively. Across these, mean P_*t*_ is 0.14 ± 0.09 pg cell**^–^**^1^ (Figure 2C). If organic P is defined as the difference between mean concentrations of P_*t*_ and P_*i*_, we determined that up to 86% of algal P may be contained within the organic phase (∼ 0.12 pg cell**^–^**^1^). Using the mean P_*t*_, we estimate the atomic C:N:P stoichiometry of glacier algal assemblages to be 1,997:73:1; however, given the difficulty in detecting cellular P, this ratio should be considered with caution.

## Discussion

Understanding the macronutrient requirements of glacier algal cells is an important first step on the way to constraining potential bottom-up controls, which may ultimately restrict bloom magnitude and extent (McCutcheon et al., 2021). The present study examined the C, N, and P content of glacier algal assemblages sampled during the mid-2019 ablation season (early August) from the southwestern margin of the GrIS, providing a direct measurement of glacier algal cellular macro-nutrient content and stoichiometry. Through this we identify deviation from standard cell-constituent:volume ratios and classic Redfield stoichiometry, and we are able to estimate glacier-algal-associated macro-nutrient reservoirs within this highly oligotrophic environment.

The absolute cellular macronutrient content of glacier algal assemblages fell within the range of values reported across a diversity of algal lineages (Montagnes et al., 1994), though they deviated from cell-constituent:volume relationships established across microalgal taxa. For example, glacier algal cellular C and N content averaged 106 ± 35 pg C cell^–1^ and 4.5 ± 1.3 pg N cell^–1^ for cell volumes that ranged ∼ 2,000–3,000 μm^3^. While absolute cellular contents were within the range reported across a host of microalgae (Montagnes et al., 1994; Finkel et al., 2016), values were conspicuously lower than would be predicted using established cell-constituent:volume ratios for cells of their size, whereby 2,000–3,000 μm^3^ cells would be expected to contain 203–304 pg C cell^–1^ and 40–62 pg N cell^–1^, respectively (Montagnes et al., 1994). Glacier algal cellular C was thus approximately half that expected based on cell volume, with cellular N an order of magnitude lower.

Laboratory and field studies have consistently identified species-level differences in microalgal elemental requirements that reflect evolutionary histories and acclimation to environmental conditions (Geider and La Roche, 2002; Finkel et al., 2016; Garcia et al., 2018). Such differences are a product of the adaptations of cellular architecture and biochemistry that correspond to changes in macromolecular composition, with protein the primary reservoir of cellular N, phospholipids, polyphosphates, and nucleic acids the reservoirs of cellular P, and cellular C largely determined by the combination of protein, lipid, and carbohydrate (Geider and La Roche, 2002; Finkel et al., 2016; Barcyte et al., 2020). In this respect, lower cellular requirements for C and N may reflect adaptation of Streptophyte glacier algae to their oligotrophic surface ice environment; documented here and in numerous previous works to be highly deficient in inorganic nutrient supplies (Table 1; Hawkings et al., 2016; Wadham et al., 2016; Holland et al., 2019). It may also reflect the lower growth rates of glacier algal communities from icy environments (∼ 5 days doubling time; Stibal et al., 2017; Williamson et al., 2018) compared to more temperate microalgal taxa (e.g., Garcia et al., 2018), whereby species with lower growth rates have associated lower protein, and thus N, content (Finkel et al., 2016). Finally, divergence from typical cell volume:constituent relationships may also reflect the highly vacuolized nature of glacier algal cells, which results in an overall higher water content compared to many other algae (Remias et al., 2009, 2012a). Separating the phylogenetic signature of microalgal elemental requirements from e.g., dynamic responses to *in situ* nutrient regimes (see below) is ideally achieved through the assessment of actively growing microalgal cells cultured under nutrient replete conditions (Geider and La Roche, 2002; Finkel et al., 2016). To date, however, an inability to culture glacier algal taxa *ex situ* has hindered advances in our understanding of their physiology and interactions with key environmental stressors (Williamson et al., 2019), necessitating field-based approaches as here.

In contrast to C and N contents, measurement of cellular P from glacier algal assemblages proved challenging, with few samples yielding detectable concentrations. This was consistent with our aqueous hydrochemistry data whereby all dissolved PO_4_^3–^-P concentrations from melted surface ice samples fell below the LoD (LoD = 4.95 μg L^–1^). For glacier algae, just four of 28 samples yielded quantifiable P, with a mean P_*t*_ content of 0.14 ± 0.09 pg P cell^–1^ across samples. To compare to published values for major marine phyla (Ho et al., 2003), a glacier algal cell volume of 2,500 μm^3^ was applied to convert to units of mmol P per liter cell volume (mmol L^–1^ cell volume), yielding ∼ 1.50 mmol P L^–1^ cell volume for our glacier algal assemblages. This is substantially lower than values reported for 15 marine eukaryote species (∼ 9.1–250 mmol P L^–1^ cell volume; see Table 2 in Ho et al., 2003), though likely reflects the nutrient deplete conditions under which glacier algal assemblages grow. For example, the cyanobacterium *Microcystis aeruginosa* demonstrated much lower cellular P content ranging from 0.2 to 1.2 pg P cell^–1^ when cultured under ambient P conditions that ranged 0–256 μg P L^–1^, with lower intracellular content associated with lower ambient P availability (Ghaffar et al., 2017). Similarly, for more closely related planktonic desmid species (Zygnematophyceae), Spijkerman and Coesel (1996) demonstrated how *Cosmarium abbreviatum var. planctonicum* originating from an oligo-meso-trophic lake was consistently capable of greater biomass production for a given amount of P (across the range 0.5–10 μmol P l^–1^) than *Staurastrum pingue*, which originated from a eutrophic lake. Cellular P content ranged 0.2–2.17 pg P cell^–1^, comparable to values reported here for glacier algae, with *C. planctonicum* shown to be an affinity species, possessing a competitive advantage in an environment where growth is permanently P limited (Spijkerman and Coesel, 1996). While limited in sample size, our data demonstrate how glacier algal macro-nutrient content broadly reflects ambient inorganic nutrient availability within surface ice habitats of the GrIS, and suggests an overall lower cellular macro-nutrient requirement likely advantageous in the ultra-oligotrophic supraglacial. These findings are consistent with recent advances in understanding potential bottom-up controls on glacier algal bloom proliferation (McCutcheon et al., 2021).

Assessment of glacier algal cellular macro-nutrient quotients allowed for a first order approximation of organic C and N stocks within assemblages inhabiting our sampling location on the marginal southwestern GrIS. Based on cellular C and N contents and abundances recorded across our samples, we calculated an average of 27.4 ± 6.0 μg C mL^–1^ and 1.2 ± 0.25 μg N mL^–1^ to be contained within glacier algal cells within our marginal sampling location. Upscaling from these point observations to the km^2^ scale (i.e., μg C or N mL^–1^ × 1.061 = kg C or N km^2^, after Williamson et al., 2018) provides an estimate of ∼ 29.0 kg C km^2^ and ∼ 1.2 kg N km^2^ stored within glacier algal assemblages inhabiting surface ice at the mid-ablation season (early August). Previously, Williamson et al. (2018) modeled glacier algal net production in southwestern Greenland forced by the number of days since snowline retreat. They estimated an overall average of 15.82 ± 8.14 kg C km^2^ produced by glacier algal assemblages across their 8.24 × 10^4^ km^2^ model region during the 2016 ablation period, with spatial variability ranging from <10 kg C km^2^ toward the equilibrium line up to ∼ 40–50 kg C km^2^ at their most marginal model regions by the end of the 2016 ablation season (Williamson et al., 2018). Our measurements of glacier algal cellular macro-nutrient content corroborate modeling estimates by Williamson et al. (2018) and extend these trends into the most marginal zone of the ice sheet, omitted by this previous study given uncertainties regarding microbial activity within such locations (Hodson et al., 2010; Stibal et al., 2012). We confirm here that glacier algal cells represent a significant producer of autochthonous organic C and N within the most marginal zone of the GrIS, likely driving the high rates of net production previously recorded for surface ice in this region (e.g., Musilova et al., 2017).

Additional to the cellular quotient of macro-nutrients, our data further demonstrate strong divergence in the relative abundance, i.e., cellular stoichiometry, of glacier algal C, N, and P relative to Redfield stoichiometry (106:16:1 C:N:P; Redfield, 1958), which we interpret here to represent the true stoichiometry of glacier algal communities under normal surface ice conditions as opposed to a dynamic response to e.g., nutrient limitation. Atomic C:N ratios of glacier algal assemblages ranged 15.6–40.8 across all samples, approximately two to six times greater than the Redfield C:N ratio of 6.6, and while P measurements proved challenging (see above), we estimate here an overall glacier algal C:N:P stoichiometry in the range 1,997:73:1. Under high light, low nutrient conditions, algal photosynthesis is often uncoupled from growth, with surplus photosynthate reallocated to storage carbohydrates and lipids that require minimal or no N and/or P inputs (Geider and La Roche, 2002; James et al., 2011; Pichrtová et al., 2014; Talmy et al., 2014). This results in a C-rich but N- and P-poor assemblage (Sterner et al., 1997), higher C:N and C:P ratios than predicted by Redfield stoichiometry, and typically conclusion that the community under study is nutrient limited (Berman-Frank, 1999; Dickman et al., 2006). For glacier algae residing within the high-light surface ice environment, it is likely that their abundant secondary phenolic pigmentation contributes to the elevated C:N and C:P ratios recorded here. This C-rich pigmentation is required to protect the cells against excess irradiance and provide a mechanism to convert abundant light energy into heat in order to liberate meltwater adjacent to the cell (Remias et al., 2012b; Dial et al., 2018; Williamson et al., 2018, 2020), likely also acting as an effective sink for excess photosynthate. However, given the characteristic oligotrophy of surface ice environments (Holland et al., 2019), and the contrasting ability of glacier algae to form widespread blooms under such conditions cryosphere wide (Williamson et al., 2019), we argue that our low absolute macro-nutrient content and elevated C:N and C:P ratios also likely signify overall reduced requirements for N and P resources by glacier algal communities, a potentially key adaptation to life in oligotrophic surface ice. This assertion is consistent with the findings of McCutcheon et al. (2021), who though concluding mineral phosphorus to be a first order control on glacier algal bloom presence, demonstrated that responses to nutrient addition (specifically phosphorous) were only evident in incubated field assemblages after 5 days of incubation, suggesting sufficient nutrient resources for glacier algal growth under ambient conditions. For context, this study reported highly comparable C:N:P stoichiometry to the present study, with POC:N:P_*org*_ ranging 690:48:1 to 2,615:196:1 across eight samples dominated by glacier algae at their main study location (McCutcheon et al., 2021). This putative lower macro-nutrient requirement would allow glacier algal assemblages to continue to progress into new oligotrophic areas of bare ice as the climate warms and the GrIS ablation zone expands into the future (Ryan et al., 2019), exacerbating local albedo decline and surface ablation in a positive feedback mechanism (Cook et al., 2020). It may also form a key consideration for ongoing efforts to culture glacier algal taxa *ex situ* under laboratory conditions.

To aid the balancing of internal stoichiometry, microalgae can actively release excess C into the ambient environment via the production and release of exopolymeric substances (EPS) (Berman-Frank, 1999; Hessen and Anderson, 2008; Palmucci et al., 2011) and/or divert excess photosynthate into the production of abundant secondary pigmentation as discussed above (Berman-Frank, 1999). For glacier algae blooming within ablating surface ice environments, EPS production has been postulated as a potentially important mechanism to aid retention of cells within the melting surface ice matrix (Williamson et al., 2019). While glacier algal EPS release remains unquantified, higher cellular C:N and C:P ratios likely impact the quality of dissolved organic matter available within surface ice environments, with consequences for other functional groups. For example, the dissolved organic nutrient phases that dominated the ambient hydrochemistry data presented here showed a DOC:DON ratio of ∼18, and a DOC:DOP ratio in the range 1,000–5,000 (Table 1). These data are highly consistent with Holland et al. (2019) who documented molar dissolved organic nutrient ratios in surface ice dominated by a glacier algal bloom further into the southwestern GrIS, with DOC:DON ranging 16–17 and DOC:DOP ranging 800–2,000. For that same 2016 glacier algal bloom, Nicholes et al. (2019) further demonstrated how bacterial secondary production was on average 28 times lower than rates of glacier algal primary production, indicating an inefficient microbial loop potentially limited by N and P resources (Holland et al., 2019; Nicholes et al., 2019). It is thus likely that glacier algal cellular stoichiometry is reflected in the quality of dissolved organic matter available within surface ice environments during blooms and may potentially limit the activity of associated communities. Constraining the short-term (within season) and long-term (inter-annual) fate of glacier algal autochthonous organic carbon and macro-nutrient production within surface ice will be the next step toward understanding their role as the dominant primary producer within supraglacial systems, and their wider impacts to biogeochemical cycling.

## Conclusion

We provide here a snapshot of glacier algal cellular macro-nutrient content and stoichiometry from assemblages sampled during the mid-ablation season at the ice margin of the southwestern GrIS. Our findings highlight comparatively lower cellular C, N, and P absolute content in glacier algal cells that deviates from standard cellular content:biovolume relationships, and high C:N and C:P ratios that likely reflect adaptation to this highly oligotrophic surface ice environment as well as the dominance of abundant secondary phenolic pigmentation within glacier algal cells. Based on these observations, we confirm the role of glacier algal blooms in the most marginal region of the ice sheet in driving autochthonous macro-nutrient accumulation, estimating that approximately 29 kg C km^2^ and 1.2 kg N km^2^ was amassed by glacier algal assemblages by the middle of the 2019 ablation season. While the long-term fate of these nutrient reservoirs remains unconstrained, our findings indicate the potential for comparatively low quality (i.e., low N and P content) dissolved organic matter release into the surface ice by glacier algal assemblages, with consequences for associated heterotrophic activity.

## Data Availability Statement

The raw data supporting the conclusions of this article will be made available by the authors, without undue reservation.

## Author Contributions

CW led the design of the study and completed all fieldwork. TT-J carried out all laboratory work. TT-J and MN performed the data analysis. All authors contributed to the manuscript.

## Conflict of Interest

The authors declare that the research was conducted in the absence of any commercial or financial relationships that could be construed as a potential conflict of interest.
